# Public health after a nuclear disaster: beyond radiation risks

**DOI:** 10.2471/BLT.15.168187

**Published:** 2016-08-30

**Authors:** Claire Leppold, Tetsuya Tanimoto, Masaharu Tsubokura

**Affiliations:** a Department of Research, Minamisoma Municipal General Hospital, Minamisoma, Fukushima, 975-0033, Japan.; b Department of Internal Medicine, Jyoban Hospital of Tokiwa Foundation, Iwaki, Fukushima, Japan.; c Department of Radiation Protection, Minamisoma Municipal General Hospital, Minamisoma, Fukushima, Japan.

In the five years since Japan’s triple disaster there has been a growth in media coverage and public interest in disaster recovery. An earthquake in March 2011 triggered a tsunami that hit the Fukushima Daiichi nuclear power plant, leading to loss of the plant’s core cooling capacities, followed by hydrogen explosions and subsequent radiation leakage. The nuclear accident is often discussed, both within Japan and abroad, from a perspective of radiation leakage – as would be expected in the aftermath of such an accident. Yet this has led to confusion about the importance of radiation risks, due to conflicting reports and a lack of awareness of ongoing problems that are unrelated to radiation. These misunderstandings deserve attention. This paper provides a brief review on post-disaster health in Fukushima prefecture, highlighting areas in need of further recognition by medical professionals and policy-makers, including the risks faced by one vulnerable population: the elderly.

The framework for understanding the health issues in post-disaster Fukushima is radiation, due to substantial amounts of radioactive material released after the nuclear accident. Although numerous studies have been published, the health risks of radiation are still not well understood, and controversy is abundant even within the realms of scientific research. No deaths or acute health effects related to radiation exposure were reported in the general public immediately after the disaster.^1^ In October 2015, the results of two studies concerning the children of Fukushima were reported within two days of each other; one found no detectable internal radiation contamination,^2^ while the other found an increased risk of thyroid cancer.^3^ Although the study reporting an increased risk of thyroid cancer was later publically criticized by the scientific community for faulty study design,^4^ this follow-up has not reached everyone and many members of the public, and even health professionals, continue to be confused by inconsistent results. This is unfortunate, in more ways than one. Controversy over radiation risk not only increases the difficulty in creating an appropriate public health response, but also diverts attention away from other post-disaster health problems that are unrelated to radiation, resulting in issues that are neglected in disaster awareness and response.

Over 80 000 people in Fukushima prefecture were forced to evacuate their homes following the nuclear accident.^5^ The event brought many changes to the affected region, including widespread social disruption through the breakdown of communities (due to the evacuation and the separation of families) and social stigma attached to being from Fukushima (largely due to incorrect assumptions about radiation exposure and risk).^1^ These social effects of the nuclear accident have been documented,^1^^,^^6^ and hold great implications for health. After the catastrophic nuclear accident in April 1986 in the city of Chernobyl in Ukraine, it was found that the increased mental health burden was the most severe of any of the post-disaster public health problems.^1^ Fukushima appears to be facing a similar situation.^1^ In addition to its impact on mental health, social disruption can be seen as a risk factor for physiological disease. Rapid life changes can lead to social isolation and psychosocial stress, which are known to be associated with poor health outcomes, including an increased risk of noncommunicable diseases. Unsurprisingly, an increase in noncommunicable disease risks, such as high blood glucose levels and hyperlipidaemia, have been found in Fukushima,^7^^,^^8^ alongside an increased burden of psychosocial distress, a phenomenon which has been described as a physical and mental health crisis.^7^ In contrast with the findings of only marginal internal radiation contamination among children and adults,^2^^,^^9^ it appears that the increasing burden of noncommunicable diseases and mental health problems may outweigh the burden of disease caused directly by radiation.

The multifaceted nature of the impact of nuclear disasters is exemplified in the issues faced by elderly residents of Fukushima. A study of 1215 elderly residents of care facilities followed up until 2013 found that those evacuated at the time of the disaster had a 3.37 times higher risk of mortality (95% confidence interval: 1.66–6.81) compared with those not evacuated; this suggests that the evacuation may have been more dangerous than the disaster itself for this population.^10^ This unexpected result illustrates the complexity of estimating disaster risks for elderly people, a challenge that has continued into the current recovery period. For example, activities such as gathering wild mushrooms in the mountains in Fukushima have been discouraged by medical workers because of radioactive contamination. This presents a different kind of risk, as stopping outdoor activities may result in increasingly sedentary lifestyles for both young and old people. In the elderly population, the effects of low levels of radiation exposure may not reach them before their death, whereas the health impact of reduced exercise may appear relatively quickly. Therefore, evacuation and lifestyle modifications to reduce radiation exposure require a clear justification; without a thorough risk assessment, these changes may be particularly harmful for vulnerable groups such as older people. In 2012, there was a discussion about the best interests of elderly people in Fukushima, with the question posed of which was more important for them: to mitigate the risk of developing cancer in 20 years’ time even if this affected their quality of life, or to continue living normal lives.^6^ This discussion continues in 2016, and there is still no clear resolution as to what should be prioritized for different population groups in the area.

Rather than strict guidelines, there are many lifestyle choices available to those who remain in Fukushima. Safety information is available through the Fukushima prefecture website: food products are strictly screened before being sold on the market and tap water has been declared safe for drinking.^11^ Foods grown in a person’s own garden are consumed at the individual’s own risk, and can go through radiation screening or not depending on personal choice. However, there is no explicit guidance about what should or should not be done in daily life. It is therefore unsurprising that medical professionals do not yet agree on how to advise older patients about continuing or abandoning old ways of living, and differing perceptions of the importance of radiation risk magnify the difficulty of this task. We argue that a risk–benefit balance in health will not be the same for every member of society. Rather than a single approach to risk management, there is a need to assess whether disaster responses such as evacuation and lifestyle modification have the same health benefit for all population groups. This cannot be done without understanding both the direct and indirect health consequences of nuclear disasters.

There is still much we can learn about the nuclear disaster in Fukushima. We urge both physicians and policy-makers to take note of the ongoing conditions in Fukushima, to better prepare for any future disasters. A clear understanding of the risks – both related and unrelated to radiation – is important for public health, to protect the health of residents and make appropriate recommendations regarding evacuation and lifestyle modifications in times of disaster. Alongside preparation for the future, local disaster recovery in Fukushima cannot take place until we know exactly what we are recovering from. While the physical damage of the earthquake and tsunami were visible, the ongoing impacts of the nuclear disaster are more elusive. This is a call for integrity in science. As doctors and scientists, we must strive to conduct high-quality research. A comprehensive perspective on Fukushima is needed, to continue the process of local disaster recovery and to improve preparation for any future nuclear disasters.
